# Defining Patient-Oriented Natural Language Processing: A New Paradigm for Research and Development to Facilitate Adoption and Use by Medical Experts

**DOI:** 10.2196/18471

**Published:** 2021-09-28

**Authors:** Abeed Sarker, Mohammed Ali Al-Garadi, Yuan-Chi Yang, Jinho Choi, Arshed A Quyyumi, Greg S Martin

**Affiliations:** 1 Department of Biomedical Informatics School of Medicine Emory University Atlanta, GA United States; 2 Department of Computer Science College of Arts and Sciences Emory University Atlanta, GA United States; 3 Emory Clinical Cardiovascular Institute, Division of Cardiology, Department of Medicine School of Medicine Emory University Atlanta, GA United States; 4 Predictive Health Institute and Center for Health Discovery and Well Being, Department of Medicine School of Medicine Emory University Atlanta, GA United States

**Keywords:** natural language processing, text mining, patient-centered care, evidence-based medicine, medical informatics

## Abstract

The capabilities of natural language processing (NLP) methods have expanded significantly in recent years, and progress has been particularly driven by advances in data science and machine learning. However, NLP is still largely underused in patient-oriented clinical research and care (POCRC). A key reason behind this is that clinical NLP methods are typically developed, optimized, and evaluated with narrowly focused data sets and tasks (eg, those for the detection of specific symptoms in free texts). Such research and development (R&D) approaches may be described as *problem oriented*, and the developed systems perform specialized tasks well. As standalone systems, however, they generally do not comprehensively meet the needs of POCRC. Thus, there is often a gap between the capabilities of clinical NLP methods and the needs of patient-facing medical experts. We believe that to increase the practical use of biomedical NLP, future R&D efforts need to be broadened to a new research paradigm—one that explicitly incorporates characteristics that are crucial for POCRC. We present our viewpoint about 4 such interrelated characteristics that can increase NLP systems’ suitability for POCRC (3 that represent NLP system properties and 1 associated with the R&D process)—(1) interpretability (the ability to explain system decisions), (2) patient centeredness (the capability to characterize diverse patients), (3) customizability (the flexibility for adapting to distinct settings, problems, and cohorts), and (4) multitask evaluation (the validation of system performance based on multiple tasks involving heterogeneous data sets). By using the NLP task of clinical concept detection as an example, we detail these characteristics and discuss how they may result in the increased uptake of NLP systems for POCRC.

## Introduction

Health informatics is an emerging interdisciplinary field that has undergone considerable evolution over recent years. This evolution has largely been driven by the availability of big data and progress in artificial intelligence, machine learning, and data science [1]. Big data from electronic health records (EHRs) have enabled researchers to train and execute neural network–based machine learning (eg, deep learning) algorithms for targeted problems, which have sometimes achieved performances that are comparable to those of human experts [2,3]. Clinical natural language processing (NLP)—one of the most complex subfields of health informatics—has also undergone rapid progress recently, which has been propelled by advanced machine learning, including deep learning [4] and text representation methods [5,6]. Clinical NLP holds particular promise for improving evidence-based, patient-oriented clinical research and care (POCRC), since significant volumes of knowledge regarding patients and research evidence are encapsulated in the form of free text [7,8]. Patient-centered medicine and patient-oriented research focus on the unique needs and characteristics of patients in addition to the specialized skills of domain experts and the best available research evidence [9-13]. Due to its emphasis on outcomes that are important to patients, the POCRC model has been suggested to be superior in terms of quality compared to disease-oriented models, which focus on surrogate end points such as laboratory measurements and physical signs [13-17]. There has therefore been a continuous push, particularly in the practice of evidence-based medicine, to promote POCRC.

NLP tools and methods are traditionally optimized and evaluated based on their abilities to perform specialized, problem-specific, site-specific technical tasks. Such methods typically lack the capabilities to go beyond the problems that they are developed for and are unable to describe the relevant diverse characteristics of individual patients or help medical experts with patient-oriented decision-making. For example, studies on the fundamental NLP task of clinical concept detection (ie, concepts from EHRs or other sources) are typically designed to detect or extract small sets of disease-specific or problem-specific homogeneous concepts and are evaluated intrinsically via metrics such as accuracy and the F-measure. Such concepts, for example, include health conditions such as obesity [18], bleeding [19], and drug reactions [20] and behavioral patterns such as tobacco [21] and alcohol [22] use. Velupillai et al [23] explained that although such systems may show high performances in intrinsic evaluation, they may have reduced value at the higher patient level. When the abovementioned problem-oriented NLP models are viewed through the lens of the well-defined model of patient-centered health care [9], they appear to be analogous to disease-oriented, evidence-based medicine models, as they focus on a particular disease or problem instead of holistically taking patients into account. Such problem-oriented NLP research and development (R&D) has resulted in the creation of state-of-the-art models for many clinical text processing tasks and is essential for incorporating NLP progress into health informatics. However, NLP methods’ inability to meet the diverse requirements of medical experts has restricted their utility in POCRC. In a clinical scenario, particularly at the point of care, it is generally unrealistic to expect medical experts to customize and use multiple complex NLP methods to fully characterize patients based on the free-text information in patients’ EHRs. As a consequence of these limitations, the transition of clinical NLP systems from their R&D environments to regular use by medical experts has been slow and limited [24,25]. By building on recent advances, clinical NLP R&D has the potential to progress from the use of disease- and problem-oriented models to the use of patient-oriented models, provided that the needs from an NLP perspective are clearly defined. The gap between the capabilities of NLP systems and the POCRC needs of medical experts may be due to the lack of specification regarding what a patient-oriented perspective for clinical NLP should comprise and how patient-oriented clinical NLP systems can complement traditional problem-oriented systems. There have been little to no formal schemes, definitions, or discussions in medical informatics literature about the aspects of patient-orientedness for NLP. Given the explosive recent advances in NLP, it is now crucial to establish the building blocks of the requirements of patient-oriented NLP, so that methodological research may be targeted to directly improve POCRC. In the following paragraphs, we attempt to formulate what aspects should be considered when developing patient-oriented NLP systems.

## Key NLP Needs for POCRC

### Interpretability as a Core System Component (Interpretability)

Recent advances in machine learning, particularly in deep learning, have resulted in their successful application to specific clinical tasks [26,27], and while most studies have relied on structured data from EHRs, some have used free-text information [4,28,29]. Some studies have even generated patient representations based on the nonlinear transformations of all encoded information in EHRs [30]. Despite the excellent results obtained by these systems in some cases, an obstacle to using these systems for POCRC—specifically when free text is involved—is the lack of interpretability. In fact, understanding how deep neural networks make their decisions is an area of active research in computer science [31,32]. Automation without interpretability means that the basis of a forecast or decision that is made by a system cannot be deciphered or explained by a medical expert. The inability to interpret the reasons behind automated systems’ decisions results in the inability of patient-facing medical experts to communicate these reasons to patients for tasks such as shared decision-making.

**Figure 1:**
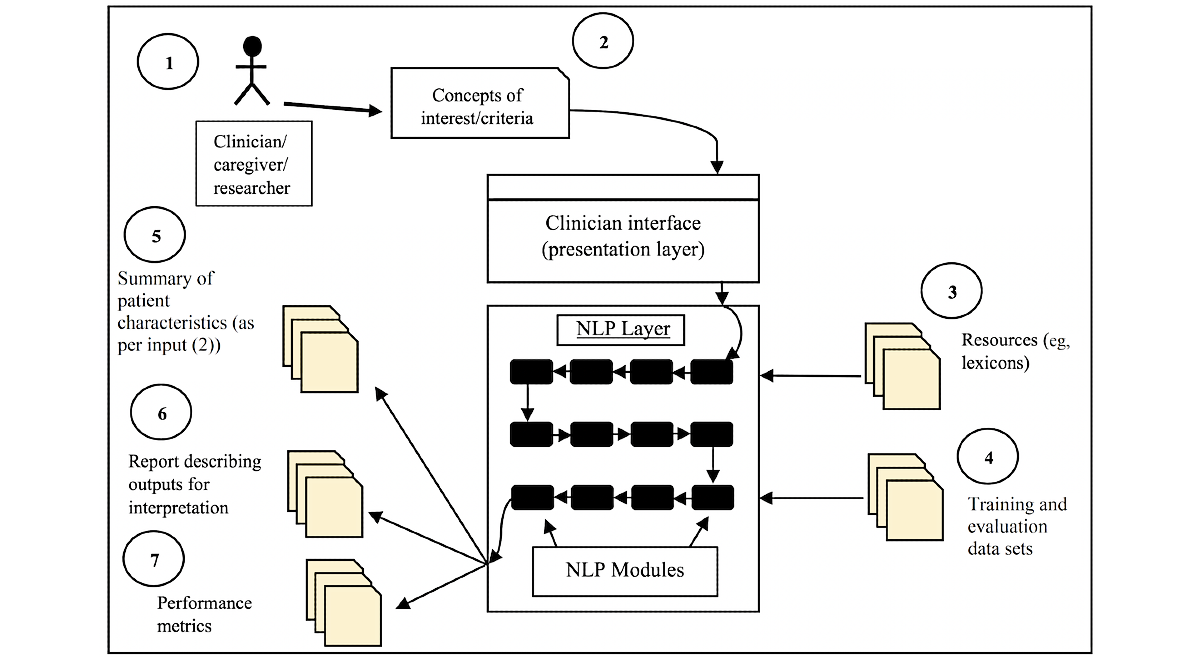
An outline of a patient-oriented NLP framework illustrating (1) the ability of the caregiver to input the required criteria via an interface that is decoupled from the technical NLP modules and (2) outputs, including reports for ensuring interpretability. NLP: natural language processing.

When designing and developing clinical NLP systems, informaticians must consider interpretability as a necessary constraint. Black-box models may be effective for a given task, but unless the decisions of a system are traceable in the desired manner, their application may not evolve beyond the problem-specific task for which they were developed [33]. One method for potentially addressing this issue is integrating reporting mechanisms with machine learning models, so that the outputs of a task are not only predictions and numeric performance metrics but also modular reports that attempt to explain the reasons behind the predictions (eg, “which span of text in the note did the system think matched with concept X?” or “what were the top features that contributed to the system’s decision?”). The hypothetical framework depicted in Figure 1 illustrates the generation of reports by a system alongside other outputs, such as performance metrics. Such reporting mechanisms are uncommon in current clinical NLP systems, as the focus of R&D is almost invariably on some type of problem-specific performance metric. This is one aspect in which involving clinical stakeholders in the development process is essential, as clinical interpretability needs may be distinct from mathematical or statistical interpretability needs [31,34].

### Broadening the Scopes of Clinical NLP Systems (Patient Centeredness)

We envision that clinical NLP systems will see greater adoption and use by medical experts for POCRC if their scopes are broader and are centered on patients rather than problems. For example, in the task of clinical concept detection, the ideal NLP systems for domain experts (and, hence, the patients they serve) would be those designed to detect ad hoc clinical concepts in free text (as specified by the expert) rather than a set of homogenous concepts. Using the current problem-oriented NLP systems perhaps adds to the burden imposed on experts, such as the burden of the “4000 clicks per shift” [35] problem, and contributes to burnout [36]. In practice, patient-oriented researchers and caregivers require a holistic view of a patient, and from the perspective of clinical concept detection, such a representation of patients requires the detection of diverse information from patients’ EHRs. Such information may range from typical concepts that past NLP research has focused on, such as diseases or symptoms, to atypical concepts such as descriptions of daily life interactions that affect the mental and physical well-being of a patient. This is perhaps the key reason why structured EHR data are preferred and are commonly used for patient-level analytical and predictive tasks. Such data present a varied set of information that, when combined, provides a detailed representation of a patient [37].

Future clinical NLP research that complements the existing advances in problem-based models should thus focus on developing frameworks that enable generalization at the patient level. For concept detection, this means enabling the specification of arbitrary clinical concepts of interest and detecting these concepts in the free-text portions of EHRs, which would result in the characterization of target patients based on these concepts. Since uncertainty is an inherent aspect of free text mining, instead of representing patient characteristics as binary variables, they can be represented by using continuous variables that represent the likelihood of a patient exhibiting specific characteristics (eg, the likelihood of viral exposure for a patient) [38]. Such a framework for concept detection can, for example, facilitate the construction of research cohorts or be used to identify eligible subjects for study enrollment based on the diverse subject information that is encoded in free text. We have seen some recent research in clinical NLP naturally evolve to take this approach to concept detection and patient characterization. For example, Stubbs et al [39] defined 13 variables, which involved diverse concepts that ranged from drug abuse to specified ranges of hemoglobin A_1c_ levels, for identifying patients who meet the selection criteria for a clinical trial. Although this approach to patient characterization via NLP was not explicitly described by the authors as *patient centered* and contrasted with typical problem-focused approaches, it represents a natural evolution toward patient-oriented NLP systems because its parameter flexibility can be used for practical tasks. Ideally, the technical complexities of the NLP algorithms for concept detection (or other purposes) should be decoupled from the interface that medical experts use, so that they may focus on specifying their patient-oriented needs (eg, ad hoc clinical concepts) without having to learn how to use multiple systems or how to execute such algorithms in multiple environments. Building NLP systems that are generalizable in such a manner is not trivial by any means, but we believe that the time is now right for designing and developing clinical NLP frameworks that incorporate such broader scopes.

### Flexible Systems Are More Likely to Stand the Test of Time (Customizability)

A problem that has been plaguing clinical NLP systems is the lack of customizability and adaptability. Many systems are so specialized to the problem-specific task for which they were designed that substantial effort is needed to adapt them to other tasks or data sources [24,40]. The complexities of most clinical NLP systems, particularly those of recent systems that involve resource-heavy language models and intricate machine learning codes (eg, systems written in TensorFlow [41]), are difficult for medical experts with non-NLP educational backgrounds to comprehend. As such, even for very similar tasks, such experts cannot customize previously developed systems to address the needs of new studies. We suspect that in most cases clinician researchers and caregivers do not even consider the possibility of diving deep into system source codes (eg, those of potentially customizable tools such as the Clinical Language Annotation, Modeling, and Processing Toolkit [42]) and customizing them according to the specific needs of a study, as they are already burdened with information overload [43].

Clinical NLP systems should thus focus on simplicity and customizability. Incorporating these aspects into the R&D of clinical NLP systems is also not trivial. However, they may be achieved by adhering to typical software development best practices. This may include using layered architectures, in which complexities are hidden under simple interfaces that expose users to customizable options. Such an architecture is shown in Figure 1. In terms of clinical concept detection, the customizability of clinical NLP systems should enable medical experts to not only specify ad hoc concepts but also tune the system for different patient-oriented tasks (eg, cohort selection) by modifying system inputs, configurations, or parameters. Improving the customizability and simplicity of clinical NLP systems will undoubtedly increase their use in POCRC.

### System Evaluations Using Multiple Data Sets With Heterogeneous Information (Multitask Evaluation)

System performance metrics obtained via evaluations based on a single data set can be misleading. Typical EHR-based free-text data sets are often constrained to small sets of patients with similar conditions, clinical settings, and social determinants, thereby causing systems that are built and evaluated based on such data sets to be overfit to the problem being studied [44]. Furthermore, the unique characteristics of the site from which the EHRs originated, such as the focus of the entity (eg, an urban children’s hospital referral center) and the educational and training backgrounds of the note writers (eg, primary care physicians vs subspecialists), also influence how free text components are written. To gauge the true performances of clinical NLP methods, including performances associated with the three previously mentioned aspects, evaluations must be conducted based on multiple data sets with differing characteristic. The reuse utility of a system is substantially diminished if it is overfit to the characteristics of a specific data set. Reporting a system’s performance metrics (eg, the F-measure for concept detection) based solely on intrinsic evaluations of such specialized data sets may also be potentially perilous, since future users may incorrectly assume that the system will exhibit similar performances on other data sets. Consequently, the evaluation of systems based on multiple data sets with distinct characteristics is imperative for ensuring the robustness of systems.

## Conclusion

**Figure 2:**
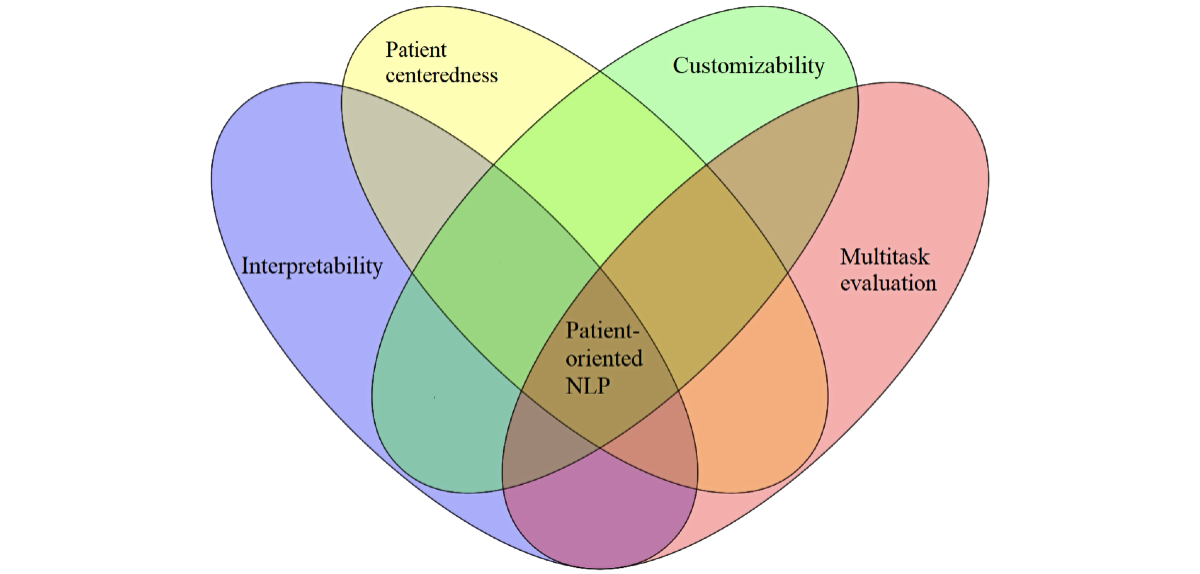
The four foundational components of patient-oriented NLP. NLP: natural language processing.

To facilitate the greater adoption of NLP in POCRC, R&D models need to build on problem-oriented approaches and transition to patient-oriented ones. In this paper, we outlined the fundamental characteristics of patient-oriented NLP system design and development. We discussed 4 interrelated factors (Figure 2) that are essential—(1) interpretability, (2) patient centeredness, (3) customizability, and (4) multitask evaluation. We believe that given the rapid recent advances in data science, it is time to initiate a new paradigm for NLP R&D—one with a patient-oriented focus—in order to increase the impact that NLP R&D has on health care. Such a paradigm shift will require overcoming many barriers, which include, but are not limited to, challenges posed by informal texts, diversities in health-related languages [24], the scarcity of annotated or labeled data, and difficulties that inhibit NLP systems’ progress from processing texts to understanding them [45]. Recent advances in NLP, such as low-shot learning [46], have the potential to aid researchers with the development of systems that are patient-oriented and, consequently, increase the impact of NLP in health care. This paradigm shift will be necessarily incremental, as researchers will build on and improve initial systems over time.

## Abbreviations

EHR: electronic health record
NLP: natural language processing
POCRC: patient-oriented clinical research and care
R&D: research and development
